# Author Correction: Interfacial design strategies for stable and high-performance perovskite/silicon tandem solar cells on industrial silicon cells

**DOI:** 10.1038/s41467-026-77840-2

**Published:** 2026-09-21

**Authors:** Lingyi Fang, Ming Ren, Biwen Li, Xuzheng Liu, Suzhe Liang, Julian Petermann, Mohammad Gholipoor, Tonghan Zhao, Johannes Sutter, Paul Fassl, Henry Weber, Ralf Niemann, Linjie Dai, Renjun Guo, Uli Lemmer, Fabian Fertig, Ulrich W. Paetzold

**Affiliations:** 1 Institute of Microstructure Technology (IMT), Karlsruhe Institute of Technology (KIT), Eggenstein-Leopoldshafen, Germany; 2 Light Technology Institute (LTI), Karlsruhe Institute of Technology (KIT), Karlsruhe, Germany; 3 School of Chemical Engineering and Technology, Sun Yat-sen University, Zhuhai, PR China; 4 Cavendish Laboratory, University of Cambridge, Cambridge, UK; 5 Eastern Institute for Advanced Study, Eastern Institute of Technology, Ningbo, PR China; 6 Hanwha Q CELLS GmbH, Bitterfeld-Wolfen, Germany

**Keywords:** Solar cells, Electrical and electronic engineering

Correction to: *Nature Communications* https://doi.org/10.1038/s41467-025-64467-y, published online 6 October 2025

In the version of this article, there were an error in the fifth paragraph of the “Experimental verification of bilayer passivation strategy” section, where in the text now reading “Specifically, the pristine perovskite film exhibits a bulk loss of 108 mV and an HTL interface loss of 27 mV, whereas the AlO_x_/PDAI_2_-treated film demonstrates a bulk loss of 112 mV and an HTL interface loss of 26 mV,” 27 mV and 26 mV originally read “4 mV” and “5 mV”; and an error in the first paragraph of the “Photovoltaic performance of tandem solar cells” section, where in the text now reading “In comparison, the PDAI_2_-based TSC achieves a PCE of 29.6%, with a *J*_SC_ of 19.48 mA cm^–2^,” 29.6% and 19.48 mA cm originally read “30.2%” and “19.89 mA cm^–2^.” There were errors in the Source Data for Supplementary Fig. 18 and associated figure plot; there were errors in Supplementary Tables 2, 3. The text, Source Data and Supplementary Information are now updated in the HTML and PDF versions of the article.

