# Supplementary material for: Interfacial design strategies for stable and high-performance perovskite/silicon tandem solar cells on industrial silicon cells
Source: Nat Commun. 2025 Oct 6;16:8881. doi: 10.1038/s41467-025-64467-y (PMC12501363; doi:10.1038/s41467-025-64467-y)
Supplement: Supplementary file 1 — Supplementary Information [file 41467_2025_64467_MOESM1_ESM.pdf]

Supplementary Information

Interfacial design strategies for stable and high-performance perovskite/silicon tandem solar cells on industrial silicon cells

Lingyi Fang[1,2], Ming Ren[3], Biwen Li[4], Xuzheng Liu[1,2], Suzhe Liang[5], Julian Petermann[1], Mohammand Gholipoor[1,2], Tonghan Zhao[1], Johannes Sutter[1,2], Paul Fassl[1,2], Henry Weber[6], Ralf Niemann[6], Linjie Dai[4], Renjun Guo[1,2]*, Uli Lemmer[1,2], Fabian Fertig[6], and Ulrich Wilhelm Paetzold[1,2]*

[1]Institute of Microstructure Technology (IMT), Karlsruhe Institute of Technology (KIT), Hermann-von-Helmholtz-Platz 1, Eggenstein-Leopoldshafen 76344, Germany

[2]Light Technology Institute (LTI), Karlsruhe Institute of Technology (KIT), Engesserstrasse 13, Karlsruhe 76131, Germany

[3]School of Chemical Engineering and Technology, Sun Yat-sen University, Zhuhai 519082, P. R. China

[4]Cavendish Laboratory, University of Cambridge, Cambridge CB3 0HE, UK

[5]Eastern Institute for Advanced Study, Eastern Institute of Technology, Ningbo 315201, P. R. China

[6]Hanwha Q CELLS GmbH, Sonnenallee 17-21, Bitterfeld-Wolfen 06766, Germany

*Email: renjun.guo@kit.edu, ulrich.paetzold@kit.edu

Supplementary Note 1

Maximum V_{OC} calculation. The maximum V_{OC} (V_{OC}^{rad}) can be calculated via equation 1 as follow[1]:

$$V_{OC}^{rad} = \Delta V_{OC}^{rad} + V_{OC}^{rad,SQA} \quad (1).$$

Here, $V_{OC}^{rad,SQA}$ represents the radiative V_{OC} limit under the Shockley–Queisser absorption model (SQA), while ΔV_{OC}^{rad} is radiative voltage deficit triggered by the trivial amount of phase inhomogeneity or segregation (sub-bandgap tails) in perovskite, which could be derived from Urbach energy.

Depending on the value of absorptivity (a_0) the ΔV_{OC}^{rad} can be estimated as follows:

$$\Delta V_{OC}^{rad} = V_{OC}^{rad} - V_{OC}^{rad,\,SQ} = -\frac{k_B T}{q}\ln\left[1 + \frac{a_0}{\frac{k_B}{E_U} - 1}\right], \quad (2)$$

where k_B is Boltzmann's constant, and T is the absolute temperature.

If a_0 is a small value:

$$q\Delta V_{OC}^{rad} \approx -\alpha_0 E_U, \quad (3)$$

here α_0 is absorption coefficient, which can be derived from a_0 according to Beer–Lambert low.

If a_0 is taken as 1:

$$\Delta V_{OC}^{rad} = \frac{k_B T}{q}\ln\left(1 - \frac{E_U}{k_B T}\right). \quad (4)$$

In our case, we approximate a_0 as 1, resulting in a calculated ΔV_{OC}^{rad} of –68.69 mV. Substituting this value into Equation (1), we obtain a V_{OC}^{rad} of 1.32 V.

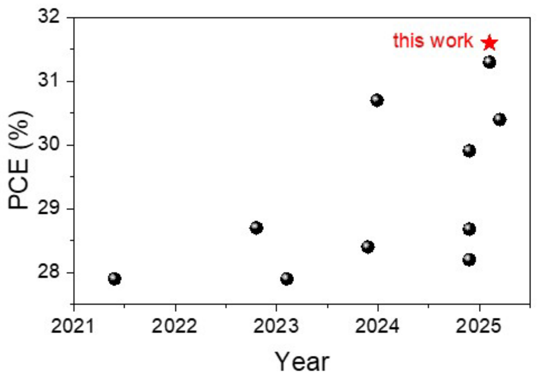

Supplementary Fig. 1 | PCE statistics of high-performance TOPCon/PERC like perovskite/silicon tandem solar cells in recent years. Citations can be found in Supplementary References 2–10.

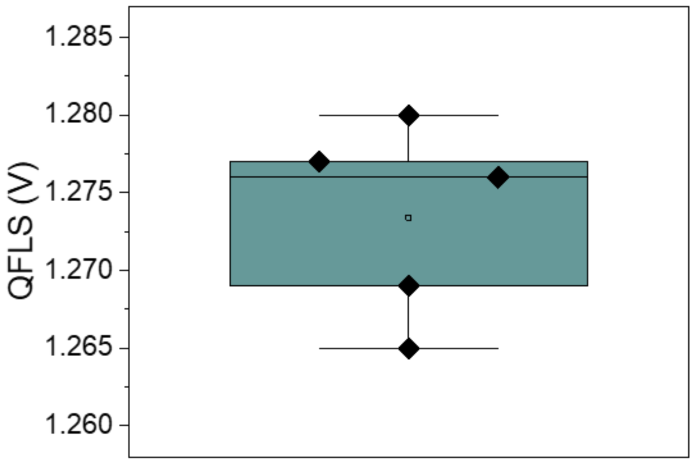

Supplementary Fig. 2 | Quasi-Fermi level splitting (QFLS) statistics for the quartz/perovskite thin films.

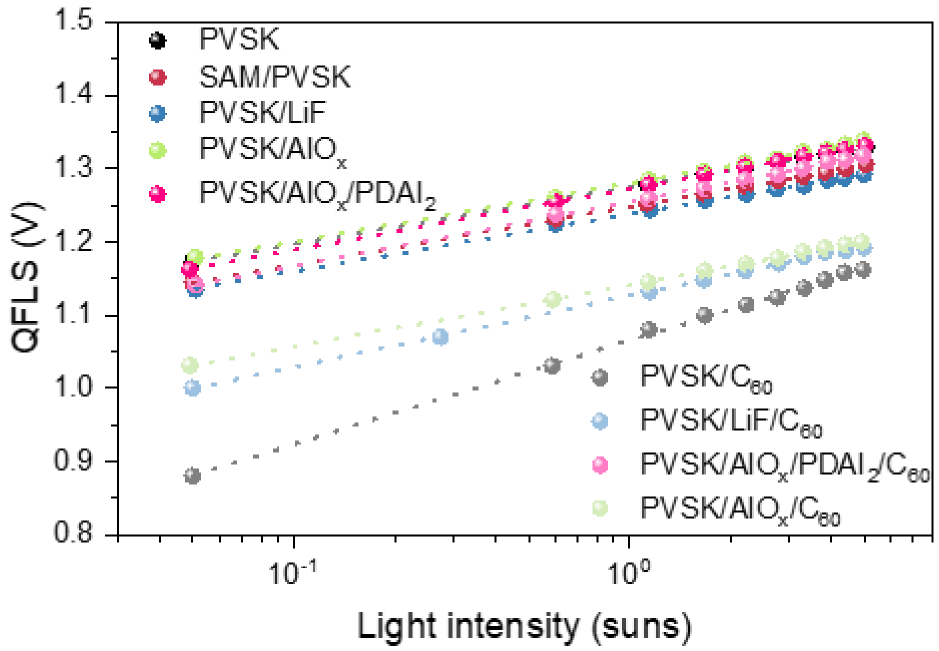

Supplementary Fig. 3 | The plots of quasi-Fermi level splitting (QFLS) as a function of light intensity for the (i) quartz/PVSK, perovskite films directly deposited onto quartz glass; (ii) ITO glass/SAM/PVSK; (iii) quartz/PVSK/C_{60}; (iv) quartz/PVSK/LiF; and (v) quartz/PVSK/LiF/C_{60} samples; (vi) quartz/PVSK/AlO_x; (vii) quartz/PVSK/AlO_x/C_{60}; (viii) quartz/PVSK/AlO_x/$PDAI_2$; and (ix) quartz/PVSK/AlO_x/$PDAI_2$/C_{60}. In the figure, substrate materials (quartz or ITO glass) are omitted from the sample.

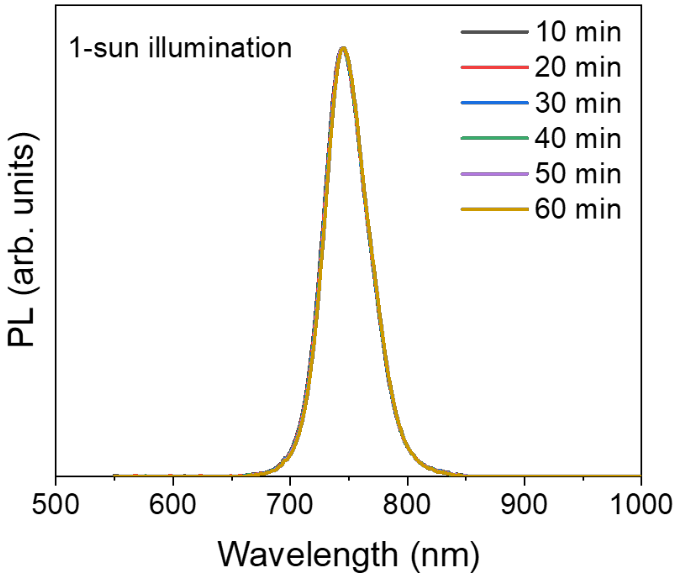

Supplementary Fig. 4 | Photoluminescence (PL) spectra of perovskite films under 1-sun illumination for 60 minutes.

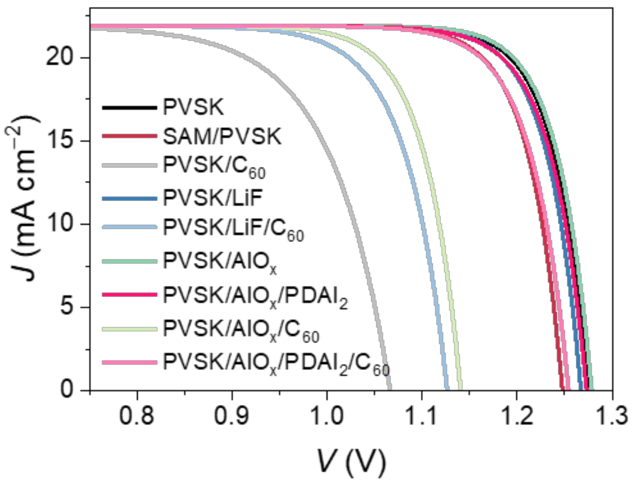

Supplementary Fig. 5 | Pseudo-*J*-*V* curves for (i) quartz/PVSK, perovskite films directly deposited onto quartz glass; (ii) ITO glass/SAM/PVSK; (iii) quartz/PVSK/C_{60}; (iv) quartz/PVSK/LiF; (v) quartz/PVSK/LiF/C_{60} samples; (vi) quartz/PVSK/AlO_x; (vii) quartz/PVSK/AlO_x/C_{60}; (viii) quartz/PVSK/AlO_x/$PDAI_2$; and (ix) quartz/PVSK/AlO_x/$PDAI_2$/C_{60}. In the figure, substrate materials (quartz or ITO glass) are omitted from the sample.

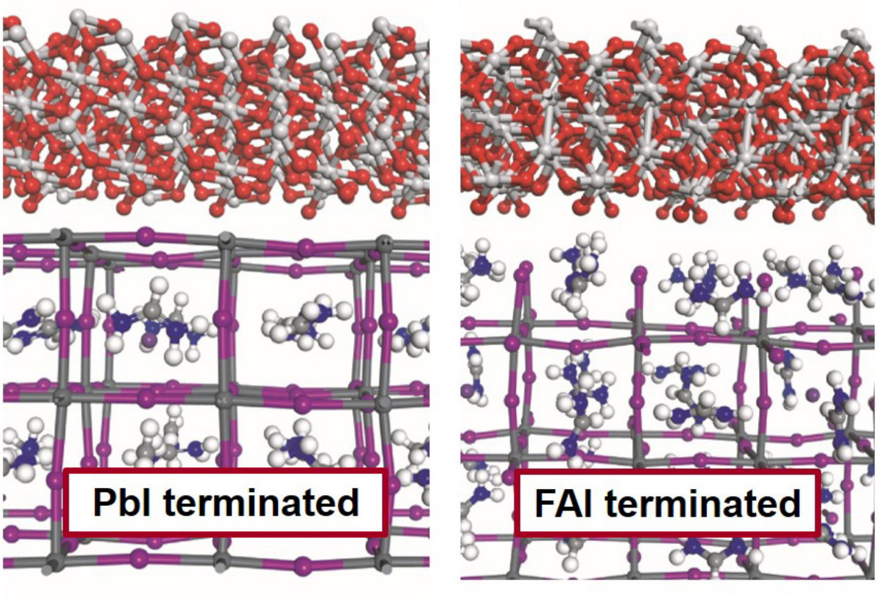

Supplementary Fig. 6 | Optimized structures of AlO_x on PbI-terminated perovskite and FAI-terminated perovskite.

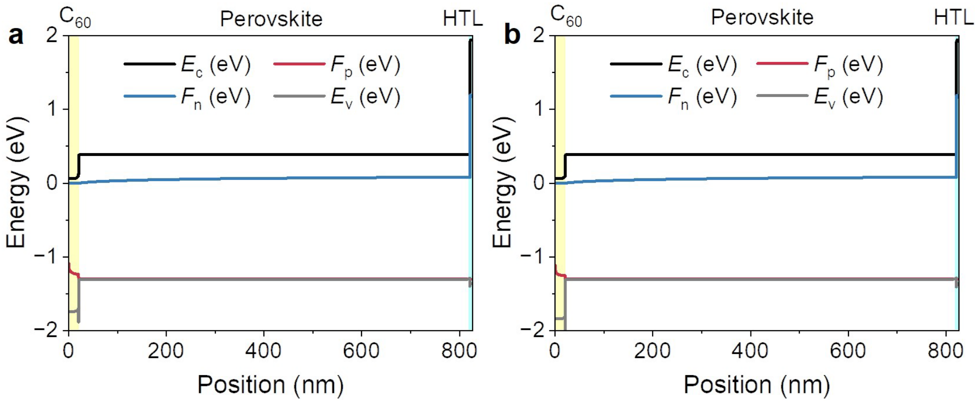

Supplementary Fig. 7 | a b, Band alignment simulated via drift-diffusion modelling for solar cells with **a,** $PDAI_2$ and **b,** AlO_x treatment. Here, E_V refers to the valence band maximum, E_C to the conduction band minimum. F_n and F_p represent electron and hole quasi-Fermi level, respectively.

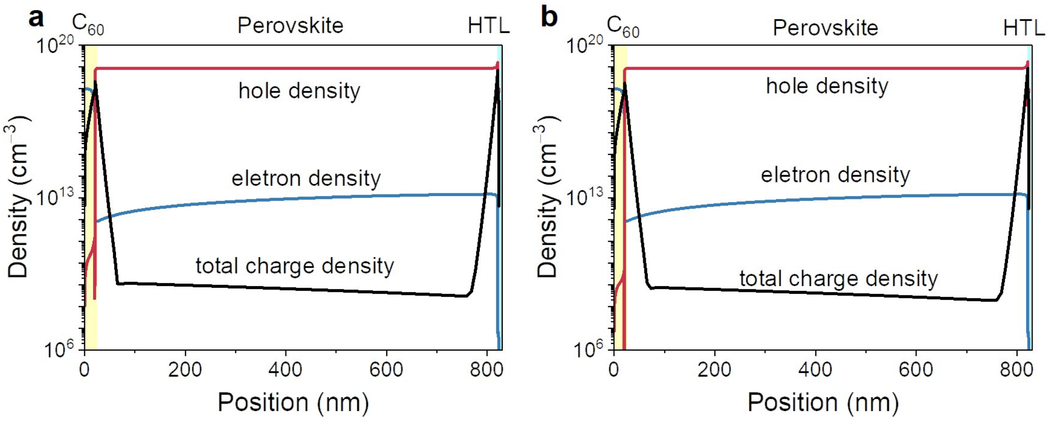

Supplementary Fig. 8 | a b, Simulated charge density profiles for **a,** $PDAI_2$- and **b,** AlO_x-treated solar cell.

Supplementary Table 1 | The device parameters for drift-diffusion simulation

Materials	Description	Parameters
ITO	Electron mobility in ITO (cm^2 V^{-1} s^{-1})	10^7
	Hole mobility in ITO (cm^2 V^{-1} s^{-1})	10^5
	Metal work function (eV)	4.7
	Relative to E_f (eV)	0.4
	Relative to E_v or E_c (eV)	0.4596
Ph-4PACz	Thickness (nm)	2
	Bandgap (eV)	3.3
	Electron affinity (eV)	2.33
	Dielectric permittivity (relative)	10
	CB effective density of state (cm^{-3})	10^{18}
	VB effective density of state (cm^{-3})	10^{19}
	Electron thermal velocity (cm s^{-1})	10^7
	Hole thermal velocity (cm s^{-1})	10^7
	Electron mobility (cm^2 V^{-1} s^{-1})	6
	Hole mobility (cm^2 V^{-1} s^{-1})	24
Perovskite	Thickness (nm)	800
	Bandgap (eV)	1.69
	Electron affinity (eV)	3.82
	Dielectric permittivity (relative)	23
	CB effective density of state (cm^{-3})	2.25×10^{18}
	VB effective density of state (cm^{-3})	1×10^{19}
	Electron thermal velocity (cm s^{-1})	10^7
	Hole thermal velocity (cm s^{-1})	10^7
	Electron mobility (cm^2 V^{-1} s^{-1})	22
	Hole mobility (cm^2 V^{-1} s^{-1})	22
	Effective mass of electrons	0.1
	Effective mass of holes	0.21
	Radiative recombination coefficient (cm^3 s^{-1})	3.16×10^{-12}

C_{60}	Thickness (nm)	20
	Bandgap (eV)	1.9
	Electron affinity (eV)	4.1
	Dielectric permittivity (relative)	6
	CB effective density of state (cm^{-3})	2.5×10^{19}
	VB effective density of state (cm^{-3})	1.8×10^{18}
	Electron thermal velocity ($cm\ s^{-1}$)	10^{7}
	Hole thermal velocity ($cm\ s^{-1}$)	10^{7}
	Electron mobility ($cm^{2}\ V^{-1}\ s^{-1}$)	20
	Hole mobility ($cm^{2}\ V^{-1}\ s^{-1}$)	0.02
NiO_x	Thickness (nm)	5
	Bandgap (eV)	3.5
	Electron affinity (eV)	1.87
	Dielectric permittivity (relative)	0.5
	CB effective density of state (cm^{-3})	10^{18}
	VB effective density of state (cm^{-3})	10^{20}
	Electron thermal velocity ($cm\ s^{-1}$)	10^{7}
	Hole thermal velocity ($cm\ s^{-1}$)	10^{7}
	Electron mobility ($cm^{2}\ V^{-1}\ s^{-1}$)	0.02
	Hole mobility ($cm^{2}\ V^{-1}\ s^{-1}$)	2
SnO_2	Thickness (nm)	20
	Bandgap (eV)	3.7
	Electron affinity (eV)	4.3
	Dielectric permittivity (relative)	10
	CB effective density of state (cm^{-3})	10^{20}
	VB effective density of state (cm^{-3})	10^{18}
	Electron thermal velocity ($cm\ s^{-1}$)	10^{7}
	Hole thermal velocity ($cm\ s^{-1}$)	10^{7}

	Electron mobility (cm^2 V^{-1} s^{-1})	0.02
	Hole mobility (cm^2 V^{-1} s^{-1})	20
Perovskite/NiO_x interface	Capture cross-section electrons (cm^2)	10^{-19}
	Capture cross-section holes (cm^2)	modified by the calculated SRV
	Total density (integrated over all energies) (cm^{-2})	modified by the calculated SRV
Perovskite/Ph-4PACz interface	Capture cross-section electrons (cm^2)	10^{-19}
	Capture cross-section holes (cm^2)	modified by the calculated SRV
	Total density (integrated over all energies) (cm^{-2})	modified by the calculated SRV
Perovskite/C_{60} interface	Capture cross-section electrons (cm^2)	10^{-19}
	Capture cross-section holes (cm^2)	10^{-17}
	Total density (integrated over all energies) (cm^{-2})	10^{11}
Silver	Electron mobility in ITO	10^5
	Hole mobility in ITO	10^7
	Metal work function (eV)	4.4
	Relative to E_f	–0.4
	Relative to E_v or E_c	–0.4749
$PDAI_2$	Thickness (nm)	1
	Bandgap (eV)	2
	Electron affinity (eV)	4.075
	Dielectric permittivity (relative)	6
	CB effective density of state (cm^{-3})	2.5×10^{19}
	VB effective density of state (cm^{-3})	1.8×10^{18}
	Electron thermal velocity (cm s^{-1})	10^7
	Hole thermal velocity (cm s^{-1})	10^7
	Electron mobility (cm^2 V^{-1} s^{-1})	20

	Hole mobility (cm^2 V^{-1} s^{-1})	0.02
AlO_x	Thickness (nm)	0.5
	Bandgap (eV)	2.5
	Electron affinity (eV)	4.065
	Dielectric permittivity (relative)	6
	CB effective density of state (cm^{-3})	2.5×10^{19}
	VB effective density of state (cm^{-3})	1.8×10^{18}
	Electron thermal velocity (cm s^{-1})	10^7
	Hole thermal velocity (cm s^{-1})	10^7
	Electron mobility (cm^2 V^{-1} s^{-1})	20
	Hole mobility (cm^2 V^{-1} s^{-1})	0.02
AlO_x/$PDAI_2$	Thickness (nm)	1
	Bandgap (eV)	2
	Electron affinity (eV)	4.05
	Dielectric permittivity (relative)	6
	CB effective density of state (cm^{-3})	2.5×10^{19}
	VB effective density of state (cm^{-3})	1.8×10^{18}
	Electron thermal velocity (cm s^{-1})	10^7
	Hole thermal velocity (cm s^{-1})	10^7
	Electron mobility (cm^2 V^{-1} s^{-1})	20
	Hole mobility (cm^2 V^{-1} s^{-1})	0.02

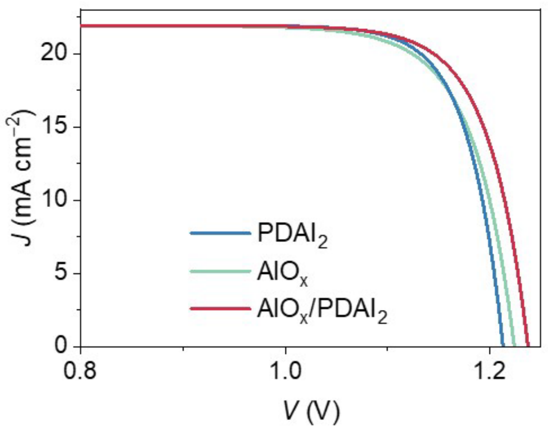

Supplementary Fig. 9 | Pseudo-J-V curves derived from intensity-dependent quasi-Fermi level splitting (QFLS) measurements of $PDAI_2$-, AlO_x-, and $AlO_x/PDAI_2$-treated perovskite films in the device stacks.

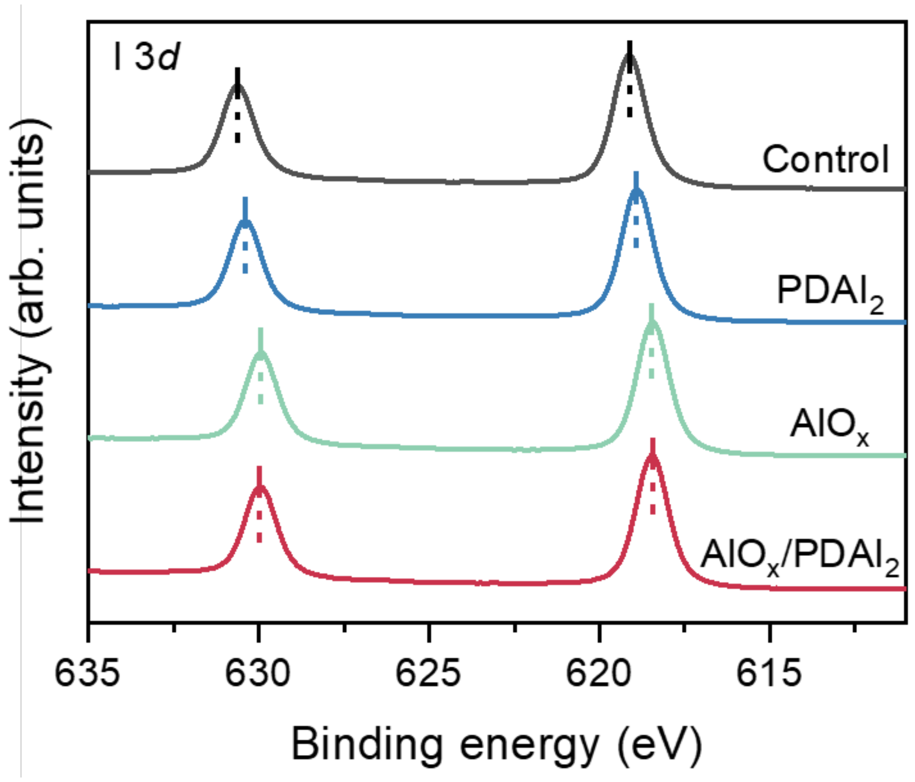

Supplementary Fig. 10 | XPS spectra of the I 3*d* core levels for the different perovskite films.

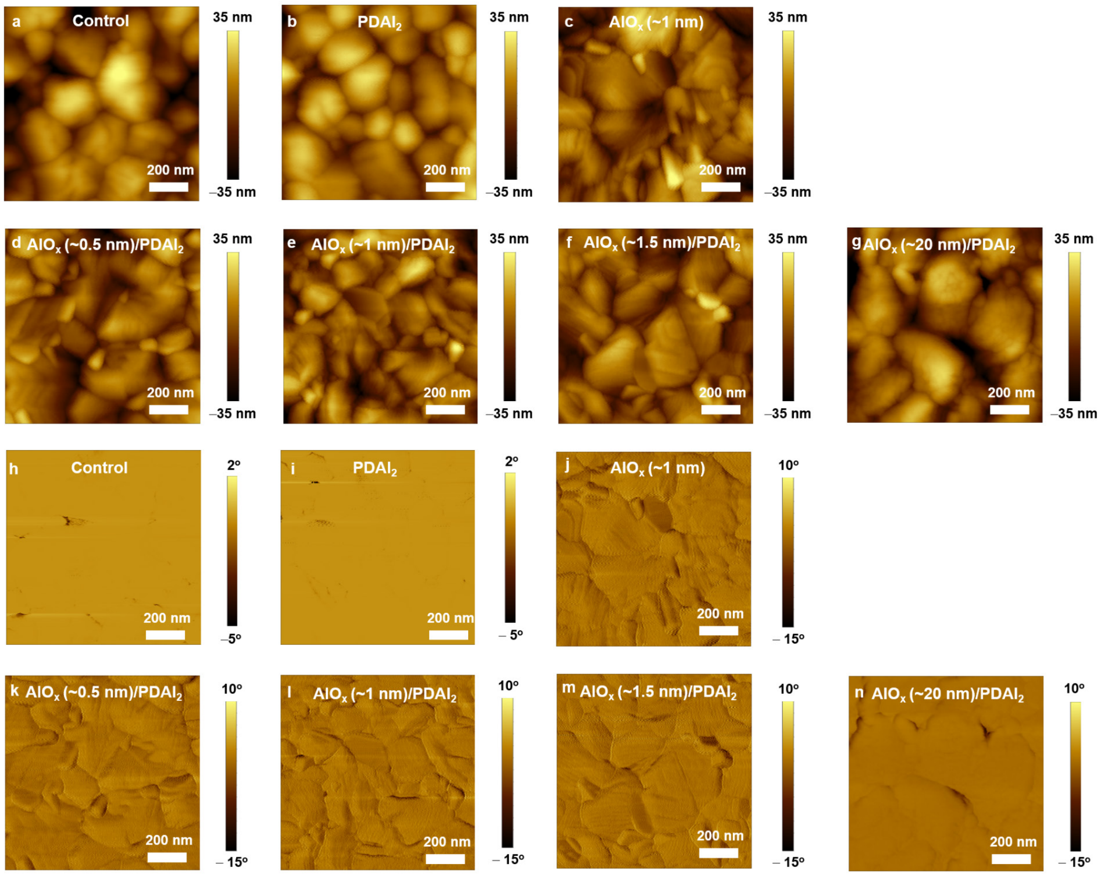

Supplementary Fig. 11 | AFM images of different perovskite films. **a-g,** Height images. **h-n,** Phase images. The size of AFM images is 1 μm × 1 μm.

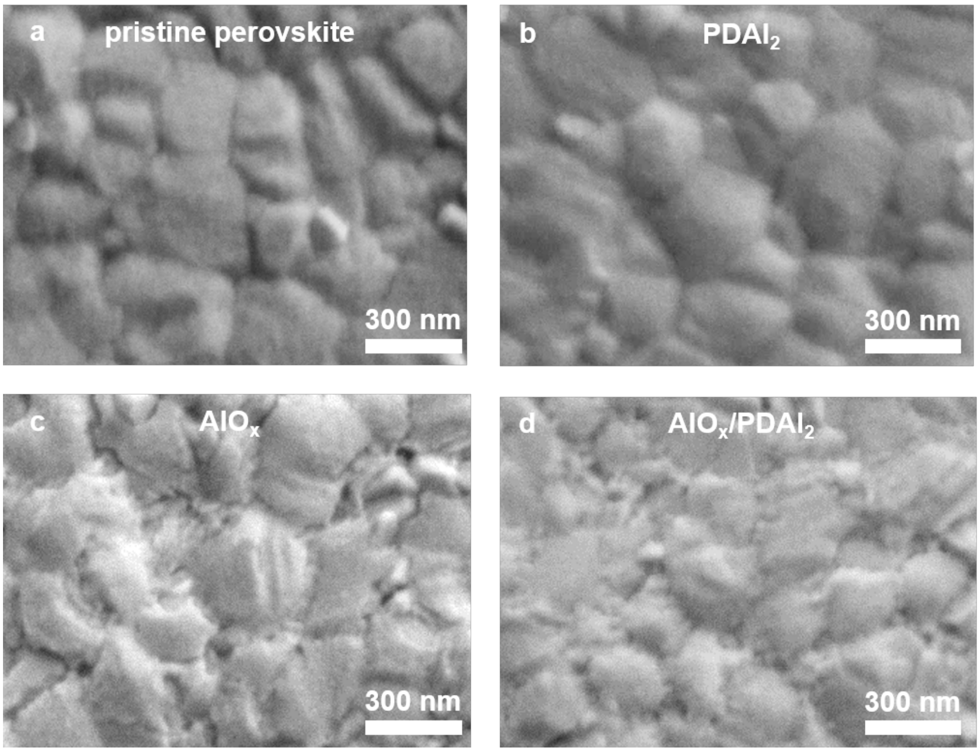

Supplementary Fig. 12 | SEM images of **a,** pristine perovskite, **b,** $PDAI_2$-treated, **c,** AlO_x-treated, and **d,** $AlO_x/PDAI_2$-treated perovskite.

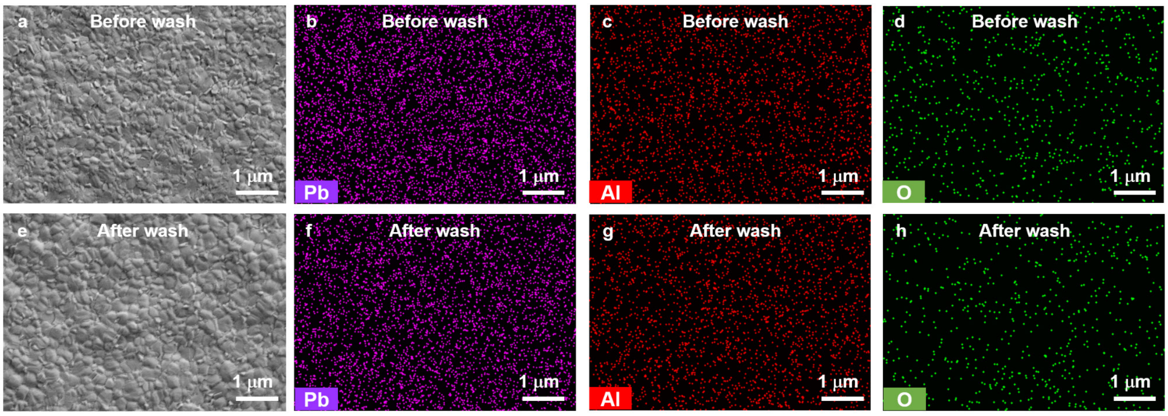

Supplementary Fig. 13 | Top-view SEM images of the $AlO_x/PDAI_2$ treated perovskite device before and after IPA washing and the corresponding EDS mapping of **b, f,** Pb element, **c, g,** Al element and **d, h,** O element.

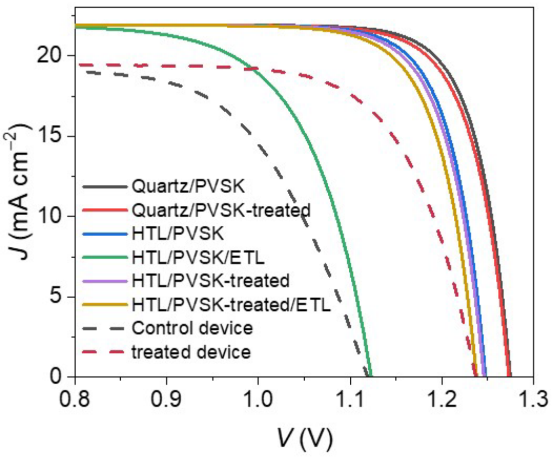

Supplementary Fig. 14 | Pseudo-J-V curves derived from intensity-dependent quasi-Fermi level splitting (QFLS) measurements of control and $AlO_x/PDAI_2$-treated perovskite films.

Supplementary Table 2 | Pseudo-J-V parameters for control and $AlO_x/PDAI_2$-treated films

	Sample	FF (%)	V_{OC} (V)	PCE (%)
Quartz/PVSK	control	87.9	1.276	24.57
	$AlO_x/PDAI_2$-treated	87.3	1.272	24.32
HTL/PVSK	control	87.5	1.249	23.95
	$AlO_x/PDAI_2$-treated	87.0	1.246	23.76
HTL/PVSK/ETL	control	80.2	1.124	19.54
	$AlO_x/PDAI_2$-treated	84.0	1.237	23.45
Device	control	76.0	1.121	17.06
	$AlO_x/PDAI_2$-treated	81.9	1.236	20.28
SQ limit	control<br>$AlO_x/PDAI_2$-treated	90.9	1.384	28.98

V_{OC}: Open-circuit voltage

FF: Fill factor

PCE: Power conversion efficiency

HTL: Hole transporting layer

ETL: Electron transporting layer

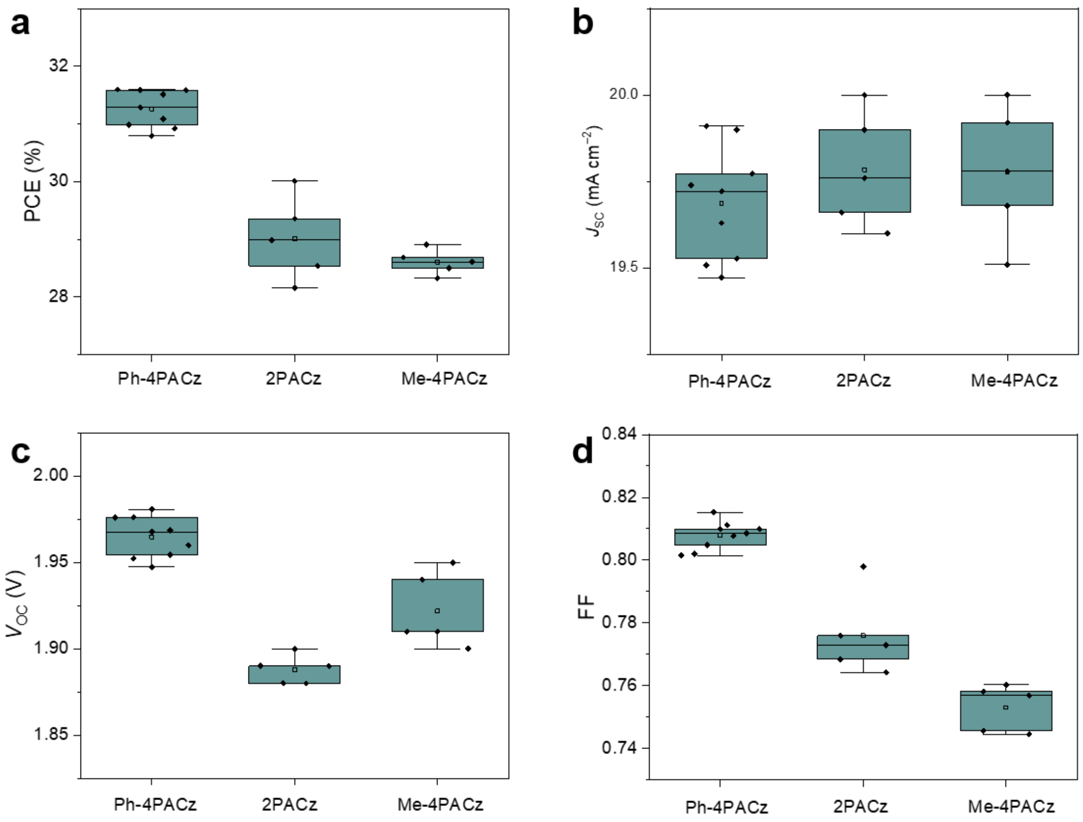

Supplementary Fig. 15 | Device performance statistics for the perovskite/silicon tandem solar cells with Ph-4PACz, 2PACz, and Me-4PACz. **a,** PCE. **b,** J_{SC}. **c,** V_{OC}. **d,** FF.

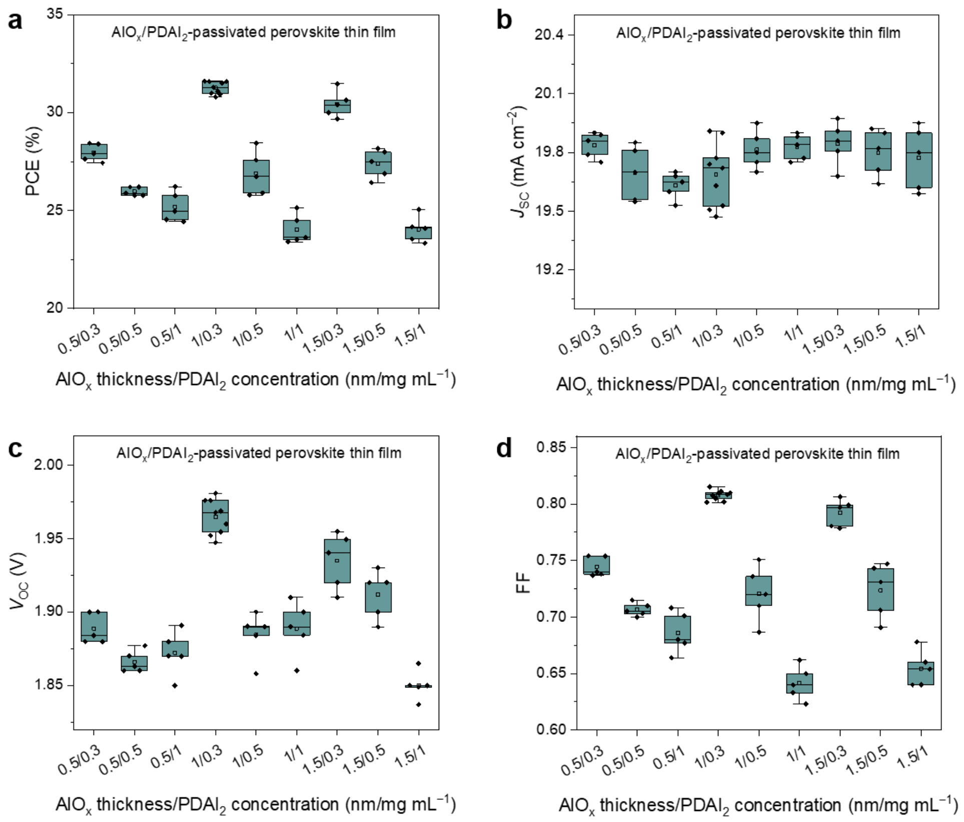

Supplementary Fig. 16 | Device performance statistics for the perovskite/silicon tandem solar cells with AlO_x/$PDAI_2$ bilayer passivation under varying AlO_x thicknesses and $PDAI_2$ concentration. **a,** PCE. **b,** J_{SC}. **c,** V_{OC}. **d,** FF.

Supplementary Table 3 | Averaged photovoltaic parameters of 9 cells measured under simulated AM1.5G illumination at an intensity of 100 mW cm^{-2}

Sample		V_{OC} (V)	FF (%)	J_{SC} (mA cm^{-2})	PCE (%)
AlO_x	reverse	1.85 ± 0.9	79.72 ± 0.11	19.90 ± 0.14	29.34 ± 0.42
	forward	1.85 ± 1.0	77.77 ± 0.15	19.89 ± 0.18	28.61 ± 0.55
$PDAI_2$	reverse	1.92 ± 0.9	79.22 ± 0.12	19.89 ± 0.18	30.25 ± 0.61
	forward	1.92 ± 1.1	78.74 ± 0.10	19.89 ± 0.22	30.07 ± 0.78
$AlO_x/PDAI_2$	reverse	1.96 ± 0.5	80.99 ± 0.12	19.91 ± 0.16	31.60 ± 0.32
	forward	1.95 ± 0.5	79.77 ± 0.17	19.95 ± 0.15	31.03 ± 0.44

V_{OC}: Open-circuit voltage

FF: Fill factor

J_{SC}: Short-circuit current density

PCE: Power conversion efficiency

測試報告

Report of Test

儀 器 名 稱<br>Device Name	Solar Cell
廠 牌 型 號<br>Model No.	Perovskite/Si Tandems
儀 器 序 號<br>Serial No.	No.19
測 試 日 期<br>Test Date	2024 / 11 / 13
送 測 單 位<br>Applicant	Karlsruhe Institute of Technology, LTI
送測單位地址<br>Applicant Address	Light Technology Institute Engesserstrasse 13 Building 30.34 76131 Karlsruhe Germany

上項儀器經本實驗室量測，結果如內文。本報告含封面及內文共 10 頁，分離使用無效。

The test device is measured by the laboratory and the results are given in the content. The report consists of 6 pages including the cover and is invalid if separated.

報告簽署人/Approved by：

報告簽署暨發行日期/Date： 2025.1.17

實驗室地址(Laboratory Address)：
高雄市路竹區路科五路 96 號 1 樓 A 區 (A area, 1F., No.96, Luke 5th Rd., Kaohsiung, Taiwan, R.O.C)

b ENLITECH Opto-Electronic CalLab

光焱科技光電校正實驗室
Enli Tech. Optoelectronic Calibration Lab.

Report No. : PVT250101

測試環境條件 Environment condition

模擬器種類 Simulator type	Steady-State Class A
照度 Irradiance	1000 W/m²
待測件溫度 DUT Temperature	25.1 °C
環境溫度 Environmental Temperature	22.7 °C

測試使用之標準件 Standard for Calibration

儀器名稱 Device Name	Reference Cell		
廠牌型號 Model No.	SRC2020		
儀器序號 Serial No.	SRC-00170		
校正機構 Cal. Laboratory	NREL		
報告編號 Report No.	2119		

標準件之溯源 Standard Traceability

儀器名稱 Device Name	Primary Reference cell		
廠牌型號 Model No.			
儀器序號 Serial No.	020033		
校正機構 Cal. Laboratory	NIST		
報告編號 Report No.			

c ENLITECH Opto-Electronic CalLab

光焱科技光電校正實驗室
Enli Tech. Optoelectronic Calibration Lab.

Report No. : PVT250101

測試結果 Test Results

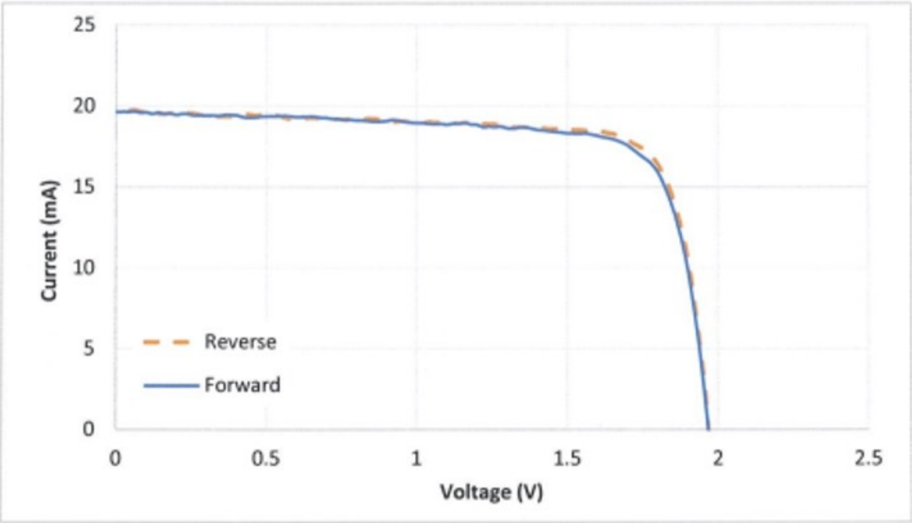

		Forward (Isc → Voc)		Reverse (Voc → Isc)	
V_{OC}	=	1968.00 ± 8.66	mV	1970.00 ± 8.67	mV
I_{SC}	=	19.62 ± 0.27	mA	19.77 ± 0.27	mA
P_{MPP}	=	29.99 ± 0.42	mW	30.77 ± 0.44	uW
V_{MPP}	=	1713.00	mV	1727.00	mV
I_{MPP}	=	17.51	mA	17.83	mA
FF	=	77.67	%	79.01	%
Efficiency	=	29.99	%	30.77	%
Aperture area	=	1.0	cm^2	1.0	cm^2

Supplementary Fig. 17 | Certification for PCE. a b, Measurement report, and **c,** device characteristics for certified perovskite/silicon tandem solar cell. The EQE spectra are not shown for compliance reasons.

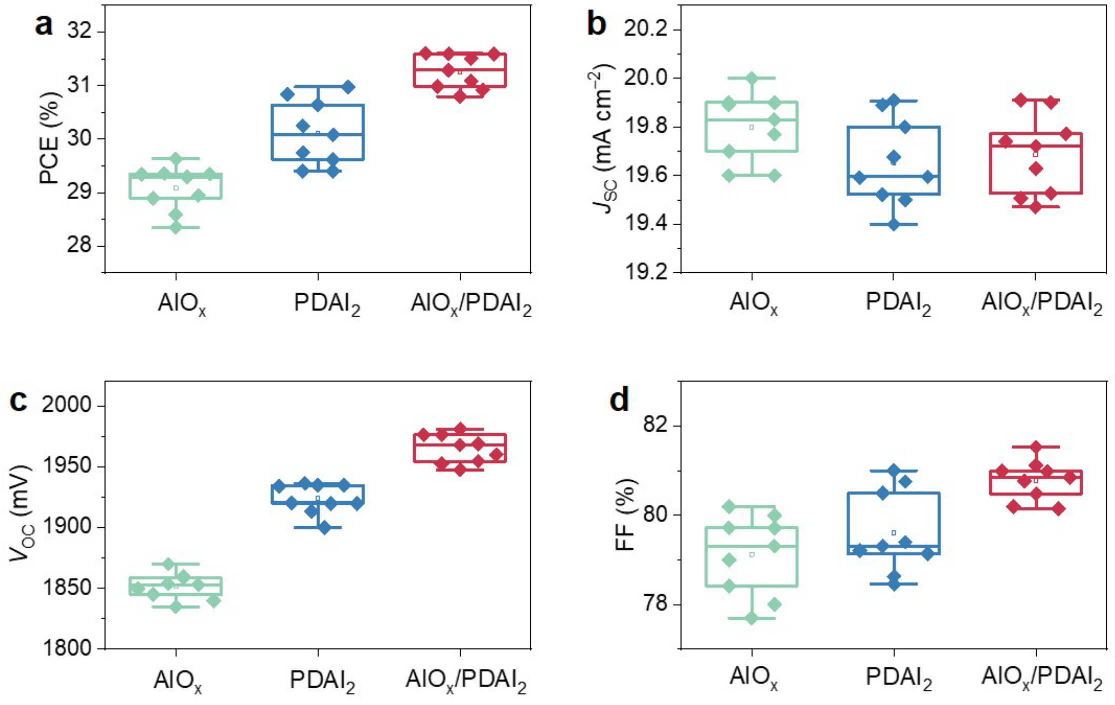

Supplementary Fig. 18 | Photovoltaic parameter statistics for perovskite/silicon TSCs with AlO_x, $PDAI_2$, $AlO_x/PDAI_2$ treatment under AM 1.5G simulated sunlight irradiation (100 mW cm^{-2}): **a,** PCE; **b,** J_{SC}; **c,** V_{OC}; **d,** FF. 9 cells of each type were tested.

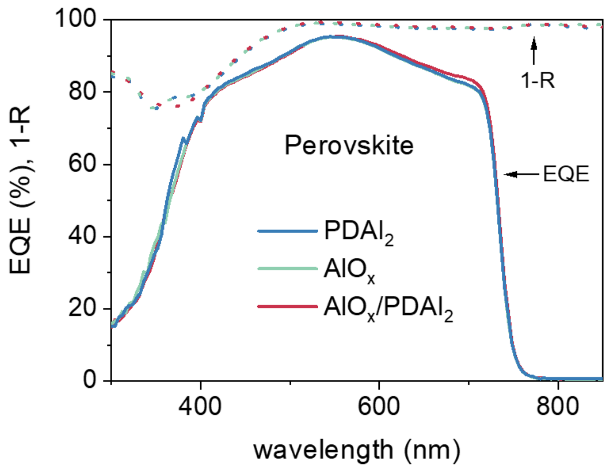

Supplementary Fig. 19 | External quantum efficiency (EQE) spectra and reflection (denoted as 1-R) of the perovskite top solar cell in the tandem solar cell. Note that the EQE of the Silicon is not shown due to the confidential information of the company.

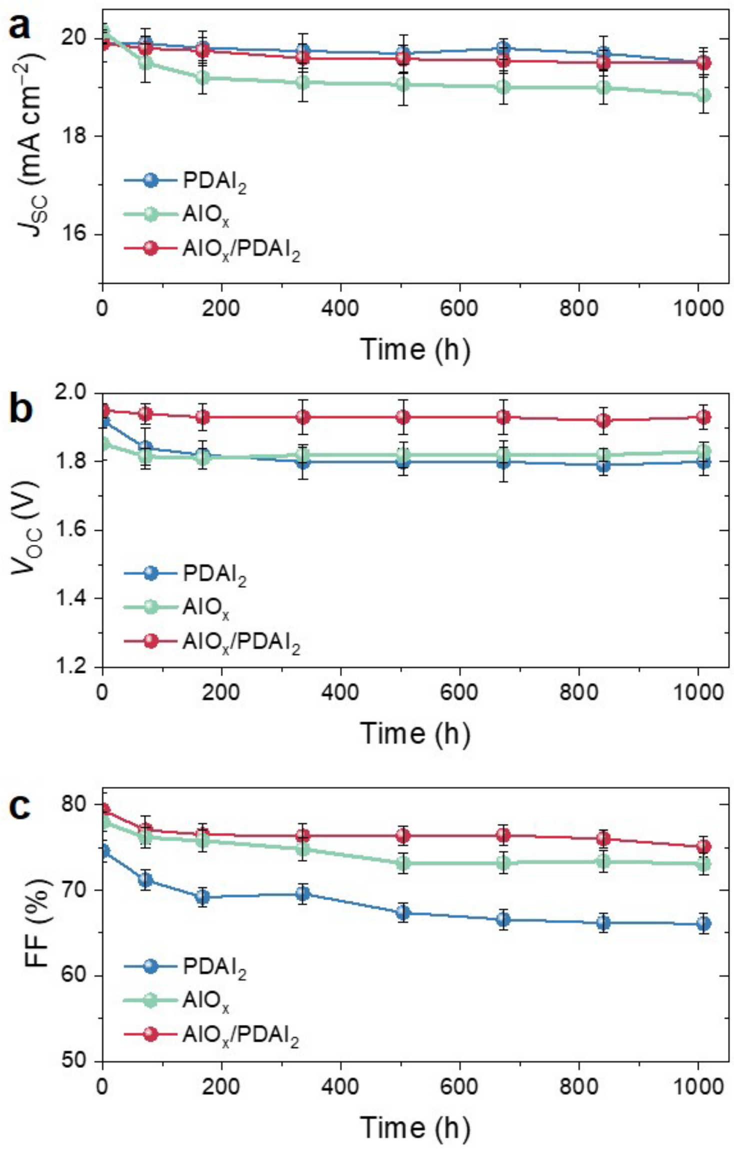

Supplementary Fig. 20 | The evolution of photovoltaic parameters **a,** J_{SC}; **b,** V_{OC}; **c,** FF in AlO_x-, $PDAI_2$-, $AlO_x/PDAI_2$-treated cells as a function of aging time at 85 ºC. Statistic data are obtained from 3 cells and the error bars represent the standard deviation.

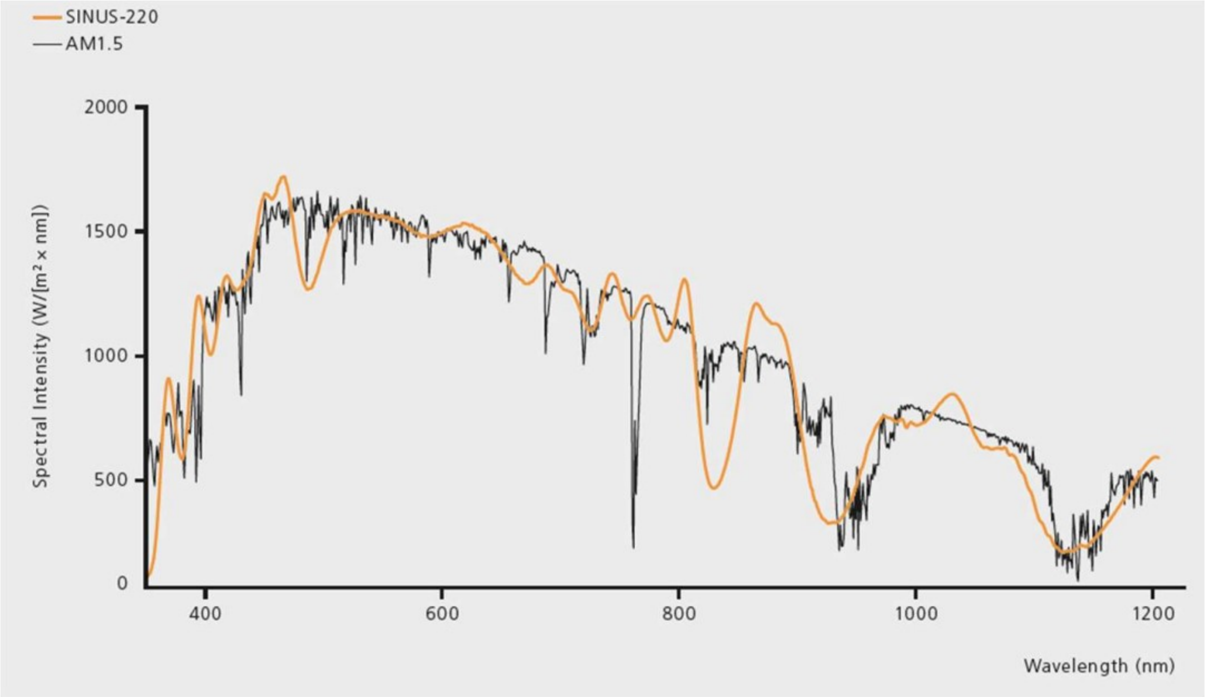

Supplementary Fig. 21 | The spectrum of WavLabs Sinus 220 compared to AM 1.5G and the total intensity between 350 and 1100 nm is around 700 W m^{-2} (equivalent to 1 sun). This LED is used as the light source for MPP tracking test.

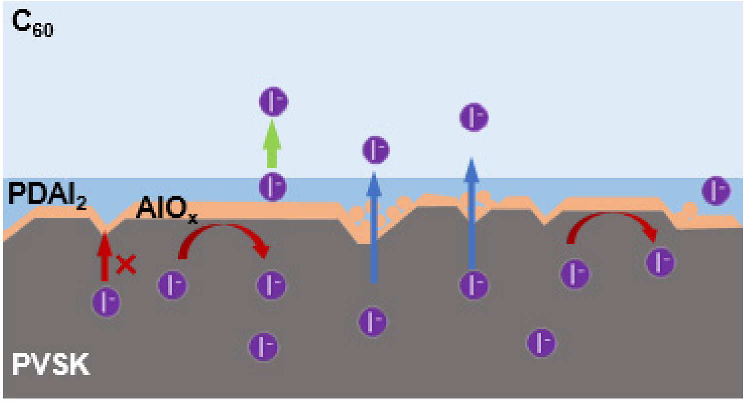

Supplementary Fig. 22 | Schematic of the proposed ion diffusion suppression by the bilayer interface. Iodide ion diffusion pathways represented by arrows of different colors. Red arrows indicate diffusion along the perovskite grain surface, which is significantly suppressed by the dense AlO_x layer. Blue arrows represent diffusion along perovskite grain boundaries, where the island-like distribution of AlO_x reduces local coverage and moderately weakens diffusion. Green arrows denote iodide ion diffusion through the $PDAI_2$ layer; since $PDAI_2$ is deposited above AlO_x, diffusion toward the C_{60} layer in this region is not hindered by AlO_x.

Supplementary References

1. Bisquert, J. Unique curve for the radiative photovoltage deficit caused by theurbach tail. *J. Phys. Chem. Lett*. **12**, 7840−7845 (2021).
2. Wu, Y. et al. 27.6% Perovskite/c-Si tandem solar cells using industrial fabricated TOPCon device. *Adv. Energy Mater.* **12**, 2200821 (2022).
3. Sveinbjörnsson, K. et al. Monolithic perovskite/silicon tandem solar cell with 28.7% efficiency using industrial silicon bottom cells. *ACS Energy Lett.* **7**, 2654-2656 (2022).
4. Luo, X. et al. Efficient perovskite/silicon tandem solar cells on industrially compatible textured silicon. *Adv. Mater.* **35**, 2207883 (2023).
5. Zhang, F. et al. Buried-interface engineering of conformal 2D/3D perovskite heterojunction for efficient perovskite/silicon tandem solar cells on industrially textured silicon. *Adv. Mater.* **35**, 2303139 (2023).
6. Qiang, Z. et al. A scalable method for fabricating monolithic perovskite/silicon tandem solar cells based on low-cost industrial silicon bottom cells. *Chem. Eng. J.* **495**, 153422 (2024).
7. Li, B. et al. Atomic-layer-deposition-free monolithic perovskite/silicon tandem solar cell reaching 29.91% power conversion on industrial PERX/TOPCon-like silicon bottom cells. *ACS Energy Lett.* **9**, 4550-4556 (2024).
8. Qiao, L. et al. Freezing halide segregation under intense light for photostable perovskite/silicon tandem solar cells. *Adv. Energy Mater.* **14**, 2302983 (2024).
9. Wang, L. et al. Highly efficient monolithic perovskite/TOPCon silicon tandem solar cells enabled by “halide locking”, *Adv. Mater.* 2416150 (2024).
10. Ye, T. et al. Molecular bridge in wide-bandgap perovskites for efficientand stable perovskite/ silicon tandem solar cells. *Adv. Funct. Mater.* 2419391 (2025).
