# Supplementary material for: Interfacial design strategies for stable and high-performance perovskite/silicon tandem solar cells on industrial silicon cells
Source: Nat Commun. 2025 Oct 6;16:8881. doi: 10.1038/s41467-025-64467-y (PMC12501363; doi:10.1038/s41467-025-64467-y)
Supplement: Supplementary file 2 — Reporting Summary [file 41467_2025_64467_MOESM2_ESM.pdf]

nature research

Corresponding author(s): Prof. Ulrich Wilhelm Paetzold

Solar Cells Reporting Summary

Nature Research wishes to improve the reproducibility of the work that we publish. This form is intended for publication with all accepted papers reporting the characterization of photovoltaic devices and provides structure for consistency and transparency in reporting. Some list items might not apply to an individual manuscript, but all fields must be completed for clarity.

For further information on Nature Research policies, including our data availability policy, see Authors & Referees.

Experimental design

Please check: are the following details reported in the manuscript?

1. Dimensions

Item	Yes/No	Details
Area of the tested solar cells	[X] Yes [ ] No	The acitive area of tested solar cells is 1.1 cm2.
Method used to determine the device area	[X] Yes [ ] No	Metal aperture mask (black color), and the aperture area of tested solar cells is 1.0 cm2.

2. Current-voltage characterization

Item	Yes/No	Details
Current density-voltage (J-V) plots in both forward and backward direction	[X] Yes [ ] No	We provide J-V in both forward and backward direction in Fig. 4b.
Voltage scan conditions<br>*For instance: scan direction, speed, dwell times*	[X] Yes [ ] No	See details in Method.
Test environment<br>*For instance: characterization temperature, in air or in glove box*	[X] Yes [ ] No	See details in Method.
Protocol for preconditioning of the device before its characterization	[ ] Yes [X] No	No preconditioning condition is used.
Stability of the J-V characteristic<br>*Verified with time evolution of the maximum power point or with the photocurrent at maximum power point; see ref. 7 for details.*	[X] Yes [ ] No	We provided MPPT data for short and long-term in Fig.4c and e

3. Hysteresis or any other unusual behaviour

Item	Yes/No	Details
Description of the unusual behaviour observed during the characterization	[ ] Yes [X] No	Negligible hysteresis is observed in the lab test but become large for the third-party certification.
Related experimental data	[X] Yes [ ] No	Reverse and forward JV scan can be found in Fig. 4b

4. Efficiency

Item	Yes/No	Details
External quantum efficiency (EQE) or incident photons to current efficiency (IPCE)	[X] Yes [ ] No	We provide EQE measurement in Fig. S19. The EQE spectra of the silicon subcell are not shown with the requirements of Hanwha Q CELLS GmbH. Hanwha Q CELLS GmbH has conflict of interests with showing EQE of bottom Si cells.
A comparison between the integrated response under the standard reference spectrum and the response measure under the simulator	[X] Yes [ ] No	We compare the integrated Jsc with one from JV scan. The difference between the integrated Jsc from EQE and Jsc from JV scan is less than 1 % difference, which is within accuracy confidence of the measurements. Integrated Jsc from EQE is slightly lower than Jsc from J-V scan.
For tandem solar cells, the bias illumination and bias voltage used for each subcell	[X] Yes [ ] No	See details in Method.

5. Calibration

Item	Yes/No	Details
Light source and reference cell or sensor used for the characterization	[X] Yes [ ] No	See details in Method.

Confirmation that the reference cell was calibrated and certified

☒ Yes ☐ No

Our solar simulator is calibrated by Si reference cell (certificated by Fraunhofer ISE).

Calculation of spectral mismatch between the reference cell and the devices under test

☒ Yes ☐ No

Spectral mismatch factor was tested by comparing the spectra of the LED solar simulator from Wavelabs and EQE measurements. This is also offered by the third-party certification.

6. Mask/aperture

Size of the mask/aperture used during testing

☒ Yes ☐ No

An optical aperture mask (1.0 cm2) is used.

Variation of the measured short-circuit current density with the mask/aperture area

☐ Yes ☒ No

No significant variations is observed.

7. Performance certification

Identity of the independent certification laboratory that confirmed the photovoltaic performance

☒ Yes ☐ No

We provide certification data in Supplementary Fig. 17.

A copy of any certificate(s)
Provide in Supplementary Information

☒ Yes ☐ No

We provide certification data in Supplementary Fig. 17.

8. Statistics

Number of solar cells tested

☒ Yes ☐ No

9 devices for each condition.

Statistical analysis of the device performance

☒ Yes ☐ No

We provide statistics in Supplementary Fig. 18.

9. Long-term stability analysis

Type of analysis, bias conditions and environmental conditions
For instance: illumination type, temperature, atmosphere humidity, encapsulation method, preconditioning temperature

☒ Yes ☐ No

We provided stability data in Fig.4d (thermal stability under 85 degree in Nitrogen) and Fig. 4e (MPPT tracking, 25 degree in Nitrogen atmosphere).
